# CXCL5‐CXCR2 signaling is a senescence‐associated secretory phenotype in preimplantation embryos

**DOI:** 10.1111/acel.13240

**Published:** 2020-09-22

**Authors:** Yuta Kawagoe, Ikko Kawashima, Yorino Sato, Naoki Okamoto, Kazuei Matsubara, Kazuhiro Kawamura

**Affiliations:** ^1^ Department of Obstetrics and Gynecology Advanced Reproduction Research Center International University of Health and Welfare School of Medicine Narita Japan; ^2^ The United Graduate School of Agriculture Sciences Iwate University Morioka Japan; ^3^ Institute of Advanced BioMedical Engineering and Science Tokyo Women's Medical University Shinjuku‐ku Japan

**Keywords:** aging, CXCL5, CXCR2, infertility, preimplantation embryo, SASP

## Abstract

Pregnancy rate of women decreases with age due to declining quality of oocytes and embryos. However, there is no established method to improve pregnancy rate in aging women. In this study, we identified a senescence‐associated secretory phenotype (SASP) factor partially responsible for the decline in embryo implantation potential. Based on microarray analysis using young and aging human embryos at the same morphological grade, 702 genes showed >fivefold increases in aging human blastocysts. Among these genes, C‐X‐C motif chemokine 5 (*CXCL5*) showed 7.7‐fold increases in aging human blastocysts. However, no‐age‐dependent changes in expression of the CXCR2, the cognate receptor for CXCL5, were found. In aging mice, *Cxcl5* transcript levels were also increased in oocytes and embryos. Treatment of young mouse embryos with CXCL5 decreased implantation rates, together with increased expression of aging markers (*P53*, *P21*, *Pai*‐*1*, and *Il*‐*6*). Moreover, CXCL5 treatment suppressed trophoblast outgrowth in young mouse blastocysts. Conversely, suppression of CXCL5‐CXCR2 signaling in aging mouse embryos using neutralizing antibodies and a receptor antagonist improved the implantation rate, leading to increases in pregnancy and delivery of normal pups. The gene expression pattern of these embryos was comparable to that in young mouse embryos showing enriched cell proliferation‐related pathways. In conclusion, we identified CXCL5 as a SASP factor in human and mouse embryos and suppression of CXCL5‐CXCR2 signaling during embryo culture improved pregnancy success in aging mice. Future analysis on CXCL5‐CXCR2 signaling suppression in human embryos could be the basis to improve embryo development and pregnancy outcome in middle‐aged infertile patients.

## Graphical abstract

We identified CXCL5 as a senescence‐associated secretory phenotype (SASP) factors in preimplantation embryos, showing elevated expression in embryos of older women and mice. The implantation rate of CXCL5‐treated young embryos from mice was decreased due to suppression of trophoblast cell proliferation. In aging embryos, suppression of CXCL5‐CXCR2 signaling during embryo culture might improve pregnancy outcome for middle‐aged women undergoing IVF.

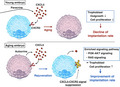

## 1 INTRODUCTION

The pregnancy rate of women declines with age. Women who are over 38 years of age showed a sharp decline in their pregnancy potential (Tan et al., 1992). During in vitro fertilization (IVF) and embryo transfer (ET) for infertile patients, pregnancy success decreased with age even when morphologically high‐quality euploidy embryos were transferred. It is considered that decline in oocyte and embryo quality leads to pregnancy failure and increases in abortion rate (Navot et al., 1991). After birth, mammalian oocytes are arrested during at prophase I until meiosis resumption triggered by the LH surge (Conti, Hsieh, Zamah, & Oh, 2012). Exposure to diverse age‐related stresses is known as one of major causes responsible for quality decline in oocytes and embryos of aging women (Cimadomo et al., 2018). Due to social and economical changes in recent years, the number of infertility patients at advanced maternal age is increasing dramatically around the world (Kocourkova, Burcin, & Kucera, 2014). Although the IVF‐ET using donor eggs from young healthy women is an established method to treat infertile aging women (Tarlatzis & Pados, 2000), many aging women would like to conceive their own genetic babies. Thus, development of new infertility treatment methods for these patients is important.

Senescence‐associated secretory phenotype (SASP) describes secretion of multiple pro‐inflammatory cytokines, chemokines, growth factors, and extracellular proteases by senescent cells (Coppe, Desprez, Krtolica, & Campisi, 2010). These factors act on cells of origin or neighboring cells through autocrine/paracrine mechanisms (Acosta et al., 2008). This phenomenon is thought to be caused by DNA damage response (DDR) that is continuously activated in senescent cells (Rodier et al., 2009). In senescent cells, increased expression of inflammatory cytokines such as IL‐6, IL‐8, TNF‐α, and TGF‐β was thought to have beneficial effects including cancer suppression and wound healing (Campisi, 2001; Demaria et al., 2014). However, it was also reported that the induced chronic inflammation by SASP factors promoted the development of aging‐related diseases (Zhu, Armstrong, Tchkonia, & Kirkland, 2014). In fact, when normal cells were cultured with senescent cells, the expression levels of aging markers including *P16*, *P21*, and some chemokines were increased together with morphological changes. Moreover, increasing senescence‐associated beta galactosidase (SA‐β‐GAL) activity was found in normal cells cultured with senescent cells (Acosta et al., 2013). Also, aging cells were shown to secret SASP factors acting through CXCR2, a chemokine receptor, to stimulates their own aging (Acosta et al., 2008). Thus, SASP factors have an aging‐stimulating effect via autocrine/paracrine actions on cells of origin or neighboring cells.

Currently, few studies have been conducted to demonstrate the relationship between SASP and the quality decline of aging germ cells. It was reported that expression levels of C‐C motif chemokine ligand 5 (*Ccl5*), one of the SASP factor, was increased in theca‐interstitial cells of aging ovary, resulting in promotion of granulosa cell apoptosis (Shen et al., 2019). This change was associated with the attenuation of preantral follicle growth, survival and estrogen secretion as well as inhibition of oocyte maturation. However, there is no study on SASP in oocytes and preimplantation embryos. In order to find SASP factors in preimplantation embryos, we analyzed differences of gene expression patterns between young and aging human embryos at the same morphological grade by using DNA microarray analysis. We selected genes highly expressed in aging embryos and identified candidate genes that might be responsible for a decline of embryo quality during aging. Among candidate genes, we found C‐X‐C motif chemokine ligand 5 (CXCL5), an inflammatory cytokine expressed in multiple types of cells, as a gene encoding for a SASP factor in aging embryos. We then demonstrated that the implantation rates of embryos were affected by regulating CXCL5 signaling mediated by its cognate receptor CXCR2 in murine embryos.

## 2 RESULTS

### 2.1 Decreases in pregnancy rate in aging patients who were transferred morphologically high‐quality blastocysts

**FIGURE 1:**
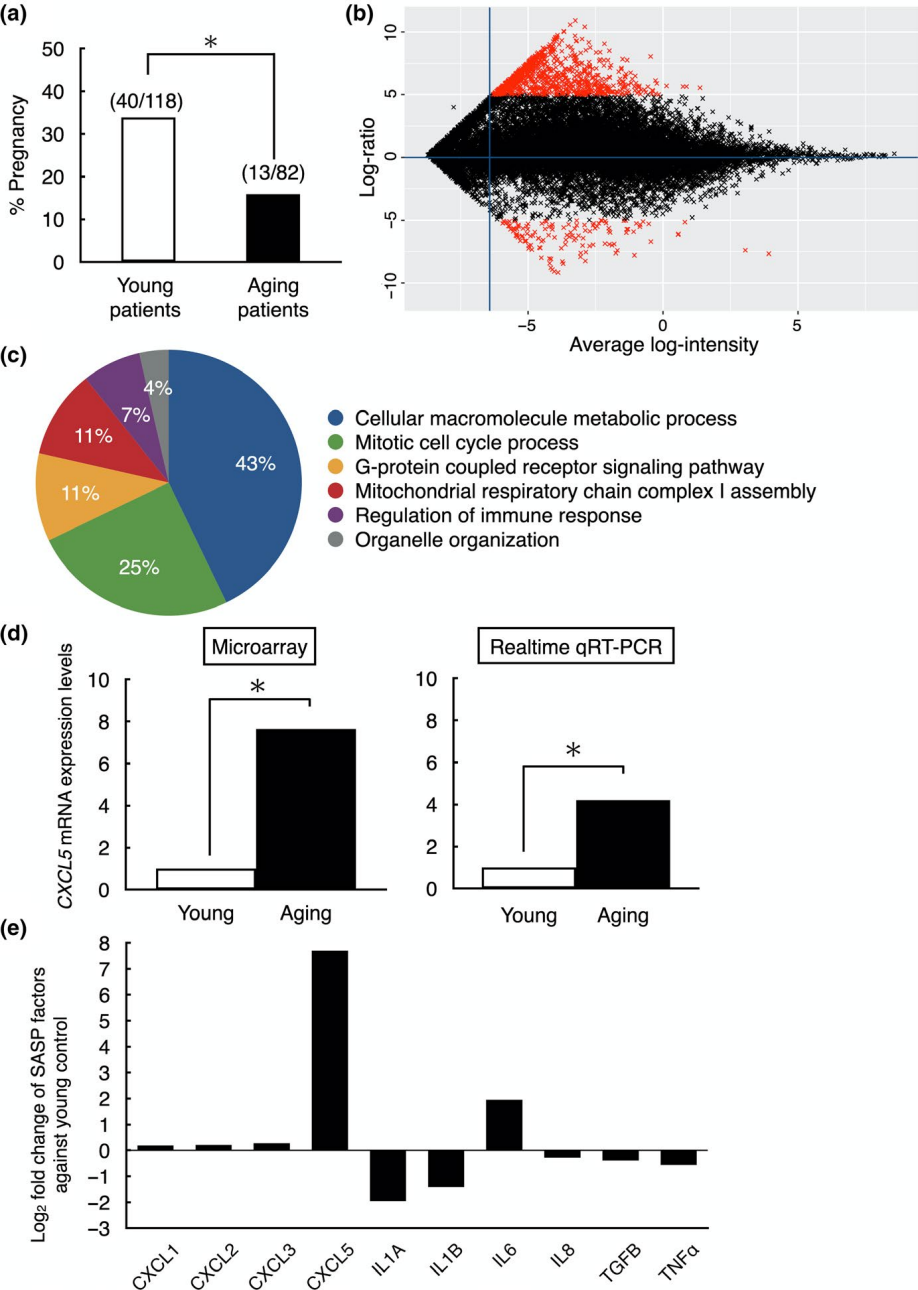
Blastocyst gene expression analysis between young and aging patients to identify SASP factors derived from human embryos. (a) Pregnancy rate of young (25–37 years, *n* = 118) and aging (38–44 years, *n* = 82) patients after high‐quality blastocyst (above 3BB by the Gardner criteria) transfer to normal thickness of endometrium (>7 mm). *p < 0.01. (b) A scatter plot of all 23,210 transcripts expressed in human blastocysts analyzed using Agilent Whole human V2 genome Oligo microarray 4 × 44 K. Gene expression in two age groups (young; 25–37 years *n* = 5, aging; 38–44 years *n* = 5) was compared. Total 820 genes were identified as differentially expressed genes (red marks, fold change >5, *p* < 0.01); 702 genes were highly expressed in aging blastocysts and 118 genes highly expressed in young blastocysts. (c) GO analysis of differentially expressed genes. Six GO terms based on biological process were significantly over‐represented in aging human blastocysts. (d) Validation of microarray data on CXCL5 expression by using quantitative real‐time RT‐PCR. *p < 0.01. Data of quantitative real‐time RT‐PCR were shown as mean ± *SE*. *n* = 3 per group. (e) Expression levels of key SASP factors in human aging blastocysts. Data were presented as fold changes against human young embryos

To confirm the influence of aging on pregnancy rates, we compared the pregnancy rates of patients at advanced ages who underwent IVF‐ET. Selected patients all had high‐quality blastocysts classified above 3BB by the Gardner criteria (Gardner, Lane, Stevens, Schlenker, & Schoolcraft, 2000) and had an endometrium thickness above 7 mm at the timing of embryo transfer. Two hundred thawed embryo transfer cycles were performed, with 53 cycles leading to pregnancy. These patients were separated into two groups based on their age: young group: patients between 25 and 37 years of age; aging group: patients between 38 and 45 years of age. Pregnancy rate was 33.9% (40/118) in the young group, whereas it was significantly decreased in aging group, 15.9% (13/82) (Figure 1a).

### 2.2 Identification of CXCL5 as a SASP factor candidate in aging human blastocysts

To identify SASP candidates, differences of gene expression levels between the young and the aging human blastocysts were determined using microarray analysis. Patient demographic data of those who donated their blastocysts were shown in Table 1. Although patient age, length of infertility and number of collected oocytes were significantly different between two groups, no difference was observed in other parameters. Five blastocysts classified above 3BB grade by Gardner criteria were used as samples in each group. A microarray platform containing about 45,000 probe sets was used, and 23,210 genes were expressed in human blastocyst samples. Data of present microarray analysis were deposited into the Gene Expression Omnibus (GEO) repository (Accession ID: GSE155476). Gene expression levels were normalized based on the expression of housekeeping genes, and differentially expressed genes were identified as log_2_ fold changes. As shown in Figure 1b, 820 genes were differentially expressed with greater than fivefold changes in aging blastocysts (*p* < 0.01); 702 genes were up‐regulated, and 118 genes were down‐regulated. All genes were plotted by statistical software package R (v. 3.5.3). In order to determine which categories of gene function were affected by aging, 820 differentially expressed genes were analyzed by gene functional analysis. As listed in Figure 1c, six GO (Gene ontology) terms involved in biological process were identified as under or over‐represented at significant levels in the aging group (FDR adjusted *p*‐value < 0.01). To find candidate SASP factors, we selected pro‐inflammatory cytokines, chemokines, and growth factors from these differentially expressed genes and then confirmed the presence of cognate receptors in the embryos.

**TABLE 1:** Patient demographic data in two age groups for microarray analysis

	Young group	Aging group
Number of donated blastocysts	5	5
Age (range)	26 (26–29)	41* (39–44)
Length of infertility (years)	5	13*
Reason for treatment	Unexplained: 1	Unexplained: 3
	Tubal factor: 4	Male factor: 1
		Tubal factor: 1
Number of stimulated cycles	4	3
Number of oocytes retrieved	21	6*

Note

Data were shown by median.

* *p* < 0.01: significant difference under Mann‐Whitney *U* test.

In the differentially expressed gene profile, we identified *CXCL5* as highly expressed in aging blastocysts. CXCL5 is a secreted factor with its cognate receptor CXCR2 expressed in preimplantation embryos (Jovanovic, Stefanoska, Radojcic, & Vicovac, 2010). To validate microarray results, the expression level of *CXCL5* in young and aging embryos were measured by using real‐time quantitative RT‐PCR. As shown in Figure 1d, the expression of *CXCL5* in microarray was increased 7.7‐fold in the aging group than that in the young group, whereas 4.2‐fold change was found using real‐time quantitative RT‐PCR. Among other major SASP factors, CXCL5 showed the highest increase in human aging blastocysts (Figure 1e).

### 2.3 Increases CXCL5 levels in oocytes and embryos in aging mice

**FIGURE 2:**
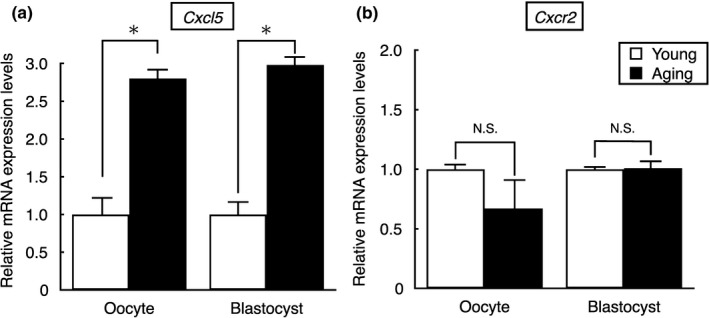
Expression of CXCL5 in oocytes and embryos of young and aging mice. (a) Transcript levels for *Cxcl5* and (b) *Cxcr2*, cognate receptor of *Cxcl5*, in oocytes and blastocysts of young and aging mice. *Cxcl5* and *Cxcr2* mRNA levels were measured by using real‐time quantitative RT‐PCR. The values of all mRNAs were normalized based on those for *Histon H2a* in the same samples. *p < 0.01, N.S.: not significant difference. All results were shown as mean ± *SE*, *n* = 3 per group

We further monitored expression levels of CXCL5 in mouse oocytes and blastocysts. In addition, expression levels of *Cxcr2*, the cognitive receptor for CXCL5, were also monitored in mouse oocytes and blastocysts. In aging mice, oocytes and blastocysts had significantly higher expression of *Cxcl5* than their young counterparts (*p* < 0.01, Figure 2a), whereas the expression level of *Cxcr2* did not change in aging oocytes and blastocysts (Figure 2b).

### 2.4 CXCL5 treatment suppressed early embryonic development of young mice for implantation

**FIGURE 3:**
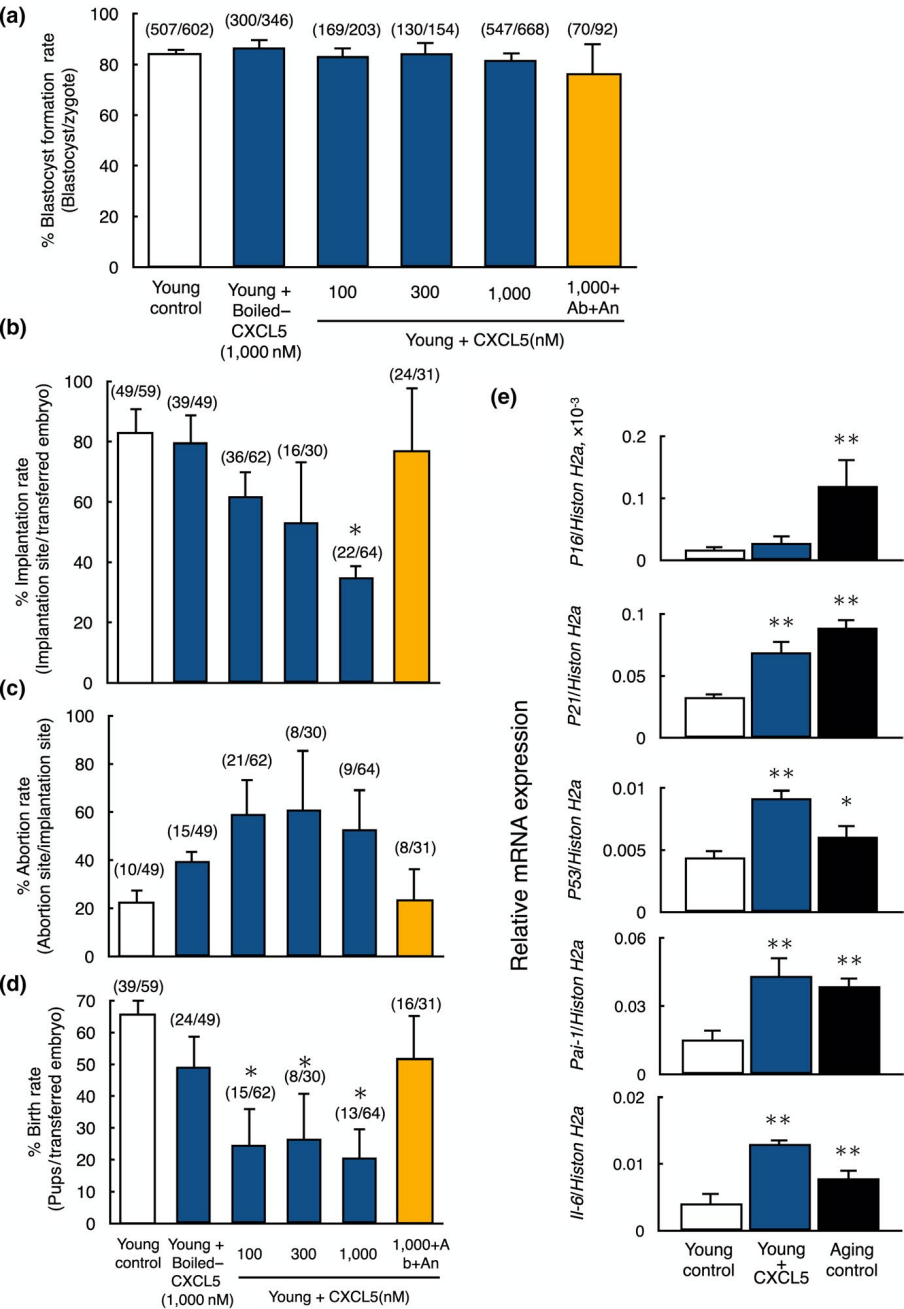
Negative impacts of CXCL5 treatment on early embryo implantation and live birth from young mice. (a) Blastocyst formation rate of young embryos treated with CXCL5 peptide. Oocyte from young mice was fertilized in vitro and allowed to develop into blastocysts with or without CXCL5 peptide treatment. The number of embryos developed to blastocyst vs. total number of embryos are listed on top of each column. Data were shown as mean ± *SE* *n* = 10–15 per group. (b‐d) Implantation (b), abortion (c), and birth rates (d) of young embryos treated with CXCL5 peptide. Cultured blastocysts were transferred to the uterus of recipient mice. On day 19.5 of pregnancy, all recipients were sacrificed to the number of pups, the implantation sites and the aborted fetuses were counted. The numbers listed on top of each column indicates the number of implantation site/total number of blastocysts transferred, the number of abortion site/the number of implantation sites and the number of pups/total number of blastocysts transferred, respectively. Data were shown as mean ± *SE* *n* = 10–15/group. *p < 0.01, vs. young control. In all experiments, boiled‐CXCL5 at 1,000 nM was served as negative control (Boiled‐CXCL5), whereas anti‐CXCL5 neutralizing antibody and CXCR2 selective antagonist were used to block CXCL5 signaling (Antibody + Antagonist). Ab, Antibody; An, Antagonist. (e) Expression levels of aging markers (*P16*, *P21*, *P53*, *Pai*‐*1*,* and Il*‐*6*) in young embryos with or without treatment with CXCL5 and in aging embryos. Levels of aging markers were measured by real‐time quantitative RT‐PCR. The values were normalized based on those for *Histon H2a* in the same sample. Data were shown as mean ± *SE*. *N* = 5–7 per group. *p < 0.05 vs. young control. **p < 0.01 vs. young control

To test effects of CXCL5 as a SASP factor on early embryo development and implantation, we treated zygotes derived from young animals with CXCL5. Although there was no difference in the blastocyst formation rate (Figure 3a), the implantation rates of CXCL5‐treated embryos were decreased in a dose‐dependent manner, with 1,000 nM CXCL5 leading to 35.0 ± 7.2% decrease (*p* < 0.01, Figure 3b). Negative controls using boiled‐CXCL5 were not significant different from untreated controls. Furthermore, cotreatment of zygotes with CXCL5 together with an anti‐CXCL5 neutralizing antibody (10 μg/ml) and CXCR2‐selective antagonist (SB225002, 10 nM) (White et al., 1998) blocked the detrimental effects of CXCL5 on implantation rates (Figure 3b). Although abortion rates were not significantly different between controls and CXCL5‐treated group (Figure 3c), birth rates of the embryos cultured with CXCL5 were significantly decreased as compared to controls (*p* < 0.05; Figure 3d).

We further monitored transcript levels for aging markers in young embryos with or without CXCL5 treatment and compared with those in aging embryos. After confirmation of elevated expression of five aging marker genes (*P16*, *P21*, *P53*, *Pai*‐*1*, and *Il*‐*6*) in blastocyst stage embryos of aging mice (Figure 3e), we compared the expression levels of these genes between young blastocysts without and with 1,000 nM CXCL5 treatment. As shown in Figure 3e, the expression levels of four aging marker genes except for *P16* were significantly increased in young embryos cultured with CXCL5 and reached levels found in aging blastocysts. Of note, although the expression levels of *P53* and *IL6* in aging human embryo were threefold and twofold higher than those in young embryos in our microarray data, respectively, there was no significant difference in the expression of *P16*, *P21*, and *PAI*‐*1*.

**FIGURE 4:**
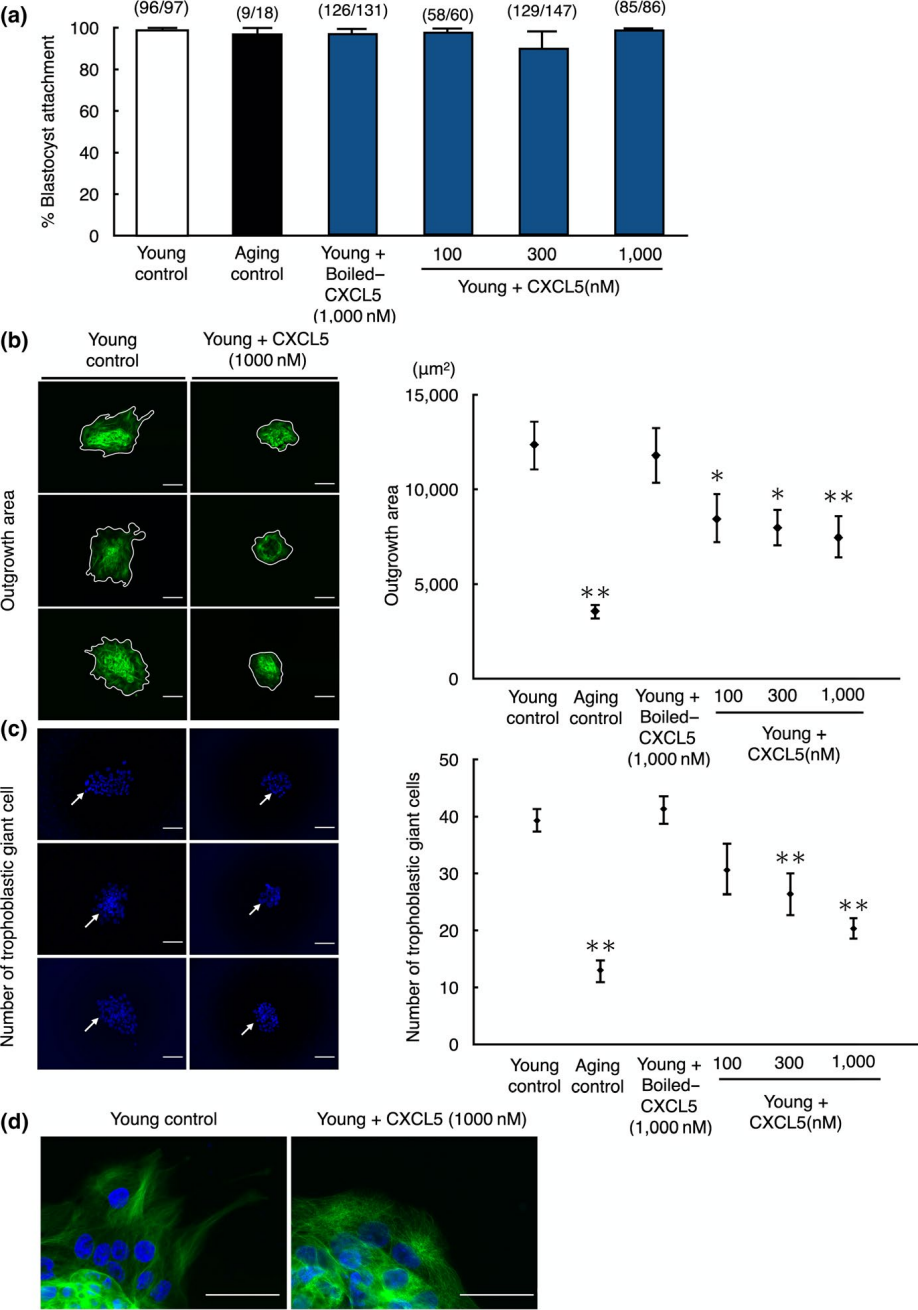
Decreases in blastocyst outgrowth without affecting blastocyst attachment in young mouse embryos by treatment with CXCL5. (a) Blastocyst attachment rate, (b) outgrowth area, and (c) number of trophoblastic giant cells of young embryos treated with CXCL5 peptide. (a) The ability of embryo attachment to culture dishes was evaluated by culturing blastocysts for an additional three days. The numbers listed on top of each column indicated the number of attached embryo/total number of blastocysts cultured. (b) Outgrowth was identified by existence of trophoblastic giant cells stained with α‐Tubulin on the culture dishes. The enclosed areas on left panels indicated outgrowth area for each group. (c) For counting trophoblastic giant cells, left panels were representative images of nuclear stained embryos. Arrows indicated trophoblastic giant cells. (d) Representative magnified images of trophoblastic giant cells at peripheral area of outgrowth for each group. Data were shown as mean ± *SE*. *N* = 5–8 per group. *p < 0.05 vs. young control. **p < 0.01 vs. young control. Boiled‐CXCL5 at 1,000 nM was served as negative control (Boiled‐CXCL5), whereas blastocysts from aging mice (Aging) were used for positive control. Bars: 100 µm

To assess the negative effect of CXCL5 on implantation potential, we carried out in vitro implantation assays. The abilities of embryo attachment and outgrowth were evaluated by comparing the young embryo group with or without CXCL5 treatment and the aging embryo groups by culturing blastocysts for an additional three days. Rate of embryo attachment was not different among the young groups without or with CXCL5, boiled‐CXCL5, and aging group (Figure 4a). However, outgrowth area visualized by α‐Tubulin staining was significantly decreased in the aging group as compared with the young group (*p* < 0.01). Furthermore, CXCL5‐treated young embryos showed a dose‐dependent decrease in outgrowth area as compared with the young group or young embryos treated with boiled CXCL5 (Figure 4b). Furthermore, the number of trophoblastic giant cells in outgrowth areas derived from embryos cultured with CXCL5 showed a dose‐dependent decline to levels comparable to the aging control as compared to the young group with boiled CXCL5 or without CXCL5 treatments (*p* < 0.01; Figure 4c). The size of CXCL5‐treated cells located at peripheral area of outgrowth was also smaller as compared with young controls (Figure 4d).

### 2.5 Suppression of CXCL5‐CXCR2 signaling pathway improved pregnancy rates of aging mouse blastocysts

**FIGURE 5:**
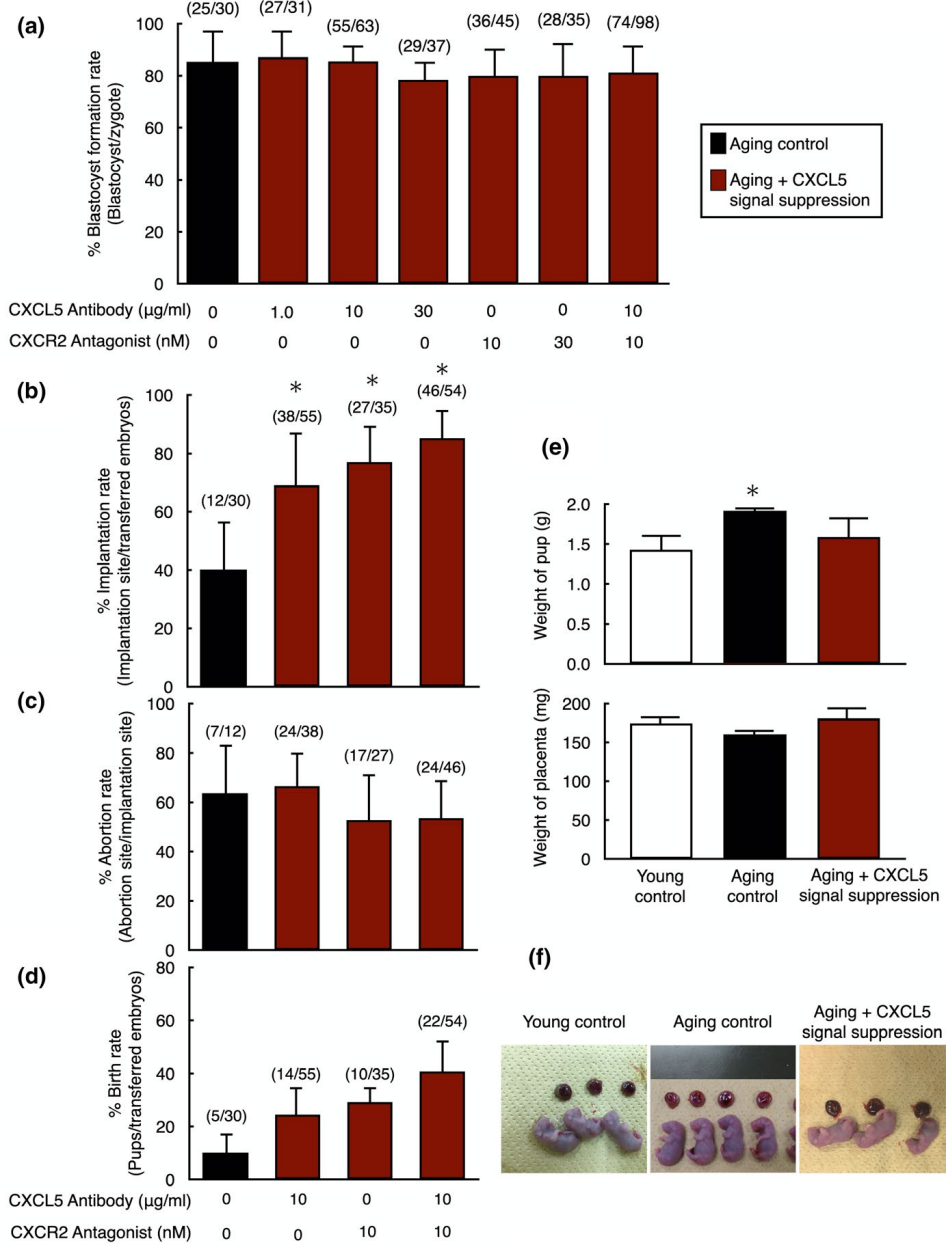
Increases in pregnancy success of aging mouse embryos with suppression of CXCL5‐CXCR2 signaling before embryo transfer. (a) Blastocyst formation rate of aging embryos with suppression of CXCL5‐CXCR2 signaling. In vitro fertilized zygotes from aging mice were allowed to develop into blastocysts without or with anti‐CXCL5 neutralizing antibody and/or CXCR2 antagonist. The number of embryos developed to blastocyst vs. total number of embryos are listed on top of each column. Data were shown as mean ± *SE*. *N* = 10–15 per group. (b–d) Implantation (b), abortion (c), and birth rates (d) of aging embryos treated with anti‐CXCL5 neutralizing antibody and/or CXCR2 antagonist. Cultured blastocysts were transferred to the uterus of recipient mice. On day 19.5 of pregnancy, the number of pups, the implantation sites, and the aborted fetuses were counted by sacrificing animals. The numbers listed on top of each column indicated the number of implantation site/total number of blastocysts transferred, the number of abortion site/the number of implantation site, and the number of pups/total number of blastocysts transferred, respectively. (e) Effects of CXCL5‐CXCR2 signaling suppression on pups and placentas derived from aging embryos. The weight of pups and placentas were measured on day 19.5 of pregnancy by sacrificing animals. (f) Representative images of pups and placentas in each group. Data were shown as mean ± *SE*. *N* = 10‐20 per group. *p < 0.01, vs. young control. Data were shown as mean ± *SE*. *N* = 10–15 per group. *p < 0.01, vs. aging control without anti‐CXCL5 neutralizing antibody and/or CXCR2 antagonist treatment

To assess whether inhibition of the CXCL5‐mediated SASP action improves pregnancy rates by increasing embryo implantation, we suppressed CXCL5‐CXCR2 signaling pathway using anti‐CXCL5 neutralizing antibody and/or a CXCR2‐selective antagonist (SB225002, 10 nM) during early embryo culture. Although suppression of CXCL5 signaling did not affect the blastocyst formation rate (Figure 5a), pregnancy rates after embryo transfer were significantly increased in groups treated with anti‐CXCL5 neutralizing antibody and/or CXCR2‐selective antagonist as compared with the aging control (*p* < 0.01). Treatment with both anti‐CXCL5 neutralizing antibody and CXCR2‐selective antagonist showed a synergistic effect to further improve pregnancy rate, leading to the same pregnancy rate as the young control group (Figure 5b). Although abortion rates were not significantly different among the aging control and the signal suppression groups (Figure 5c), birth rates were increased when CXCL5 signal was suppressed. In the case of CXCL5 signal suppression by using both anti‐CXCL5 neutralizing antibody and the CXCR2‐selective antagonist, the birth rate was significantly increased as compared to the aging control (*p* < 0.01; Figure 5d). To assess whether CXCL5 signal suppression has any unwanted side effects, young embryos were treated with anti‐CXCL5 neutralizing antibody and/or the CXCR2‐selective antagonist to suppress endogenous CXCL5‐CXCR2 signaling. Blastocyst formation and pregnancy rates were not significantly different between control and CXCL5 signaling‐suppressed groups in young animals (Figure S1A,B). Both abortion and birth rates were also not influenced by CXCL5 signal suppression (Figure S1C,D).

To assess the influence of signal suppression on pup health, the weights of pups and placentas were measured. Although weights of pups from the aging group were significantly heavier as compared to the young control likely due to reduced number of growing fetus in uterus, the weight of pups from the group of CXCL5 signal suppression was identical to young control (Figure 5e). Weights of placentas showed no significant differences between the three groups (Figure 5e). In addition, gross morphology of pups and placentas was not different among all groups (Figure 5f).

### 2.6 Similarity of gene expression pattern between young and CXCL5‐CXCR2 signaling‐suppressed aging blastocysts

**FIGURE 6:**
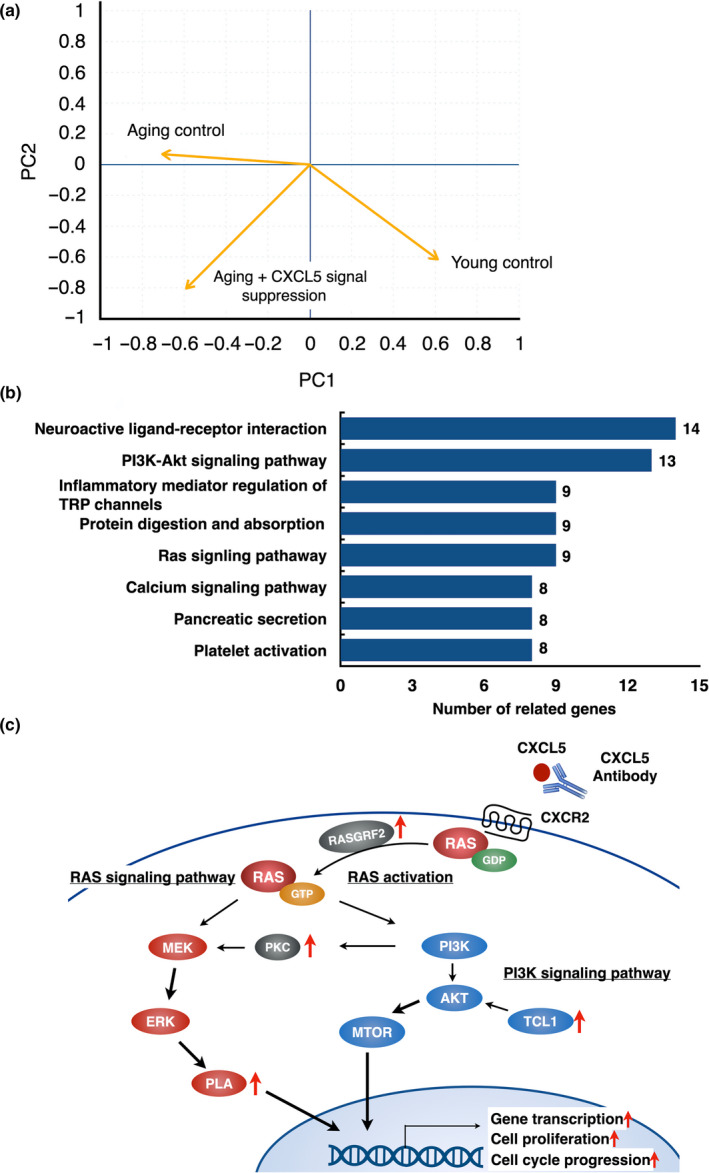
Analysis of differentially expressed genes between aging mouse blastocysts with and without CXCL5 signaling suppression. (a) Similarity of gene expression pattern of embryos between young and aging mouse blastocysts with or without CXCL5 signaling suppression. Using blastocysts samples, principal component (PC) analysis using data of microarray analysis was performed. (b) KEGG (Kyoto Encyclopedia of Genes and Genomes) pathway analysis on differentially expressed genes in aging embryos with or without CXCL5 signal suppression using data of microarray analysis. The graph showed the enriched pathways. Numbers listed on left of each column indicate the number of related genes. (c) Schematic diagram of enriched signaling pathway following suppression of CXCL5 signaling. PI3 K‐AKT and RAS signaling pathway were affected by CXCL5 signaling suppression. The genes located besides the red arrows are enriched and significantly up‐regulated in CXCL5‐suppressed aging blastocysts

To compare gene expression patterns among young, aging and CXCL5‐CXCR2 signaling‐suppressed aging blastocysts, microarray analysis was performed. The results of these microarray data were deposited into GEO repository (Accession ID: GSE155477). Each experimental group was clustered by their gene expression pattern. As shown in Figure 6a, gene expression pattern of CXCL5‐CXCR2 signaling‐suppressed aging blastocysts was closer to that of the young blastocysts as compared with aging blastocysts.

### 2.7 Increase in the expression of key cell proliferation genes in aging blastocysts with CXCL5‐CXCR2 signaling suppression

Signaling pathway analysis was performed to identify changes in expression of signal pathway genes in cultured blastocysts following CXCL5‐CXCR2 signaling suppression. Differentially expressed genes between aging blastocysts and CXCL5‐suppressed aging blastocysts were analyzed by using KEGG enrichment analysis. Eight signaling pathways were significantly enriched by CXCL5 suppression (Figure 6b). Among them, cell proliferation‐related pathways, such as “PI3 K signaling pathway” and “RAS signaling pathway,” were significantly enriched in CXCL5‐suppressed blastocysts (Figure 6c).

## 3 DISCUSSION

Based on comprehensive gene expression analysis, we identified *CXCL5* as an aging‐associated gene in early human embryos and found that CXCL5 treatment led to quality decline of young mouse embryos, resulting in implantation failure. Furthermore, we also found that suppressing CXCL5‐CXCR2 signaling of aging mouse embryos improved pregnancy success.

Among SASP candidates, *CXCL5* showed the highest expression levels in aging human embryos expressing its cognate receptor, *CXCR2*. Other major SASP factors known to show increased expression in various aging tissues and organs, including liver, kidney, lung, and adipose (Ghosh, O'Brien, Mau, Qi, & Yung, 2019; Melk et al., 2004; Rubini, 2013), were not increased as high as CXCL5 in human embryos. Although we could not measure secreted CXCL5 peptide in human embryo culture medium due to limited amount of materials, our animal data suggested that CXCL5 likely acted on embryos via autocrine/paracrine fashions. There are few studies on SASP factors in reproductive medicine and physiology. In aging ovary, the expression level of C‐C motif chemokine ligand 5 (*Ccl5*), one of the SASP factors, was increased in theca‐interstitial cells, resulting in suppression of preantral follicle growth and oocyte maturation (Shen et al., 2019). However, based on our best knowledge, no report regarding a SASP factor related to aging of oocytes/embryos has been published.

Senescence‐associated secretory phenotype factors are known to increase in diverse cells and tissues during aging and act on their cognitive receptors, resulting in activation of inflammatory signaling pathways to promote cellular senescence (Zhu et al., 2014). In our study, we found that CXCL5 promoted cellular senescence in young mouse embryos with increases in aging marker genes, resulting in implantation failure by inducing suppression of cell proliferation in trophoblast cells. In other types of cells and tissues, CXCL5 was shown to act as a SASP factor. CXCL5 expression was increased during tumorigenesis and metastasis in different types of cancer, including breast, lung, pancreatic, and prostate (Arenberg et al., 1998; Kuo, Chen, Chen, Shen, & Hsu, 2011). Furthermore, CXCL5 treatment decreased proliferation of prostate stromal fibroblasts (Begley, Kasina, MacDonald, & Macoska, 2008). These data support our findings on promotion of cellular senescence by CXCL5 in young embryos. Suppression of autocrine/paracrine actions of SASP factors is expected to prevent deleterious events associated with cellular senescence. In this study, we demonstrated that suppression of CXCL5‐CXCR2 signal improved the implantation rate of aging mouse embryos with increased expression of key cell proliferation genes. Of interest, gene knockdown of *CXCR2* by shRNA to suppress CXCL5‐CXCR2 signaling prevented aging‐associated decline of cell proliferation and extended cellular lifespan in human embryonic lung fibroblasts (IMR‐90, WI‐38) and human mammary epithelial cells (Acosta et al., 2008). In addition, suppression of other SASP factors such as IL‐1α, IL‐6, IL‐8, and GROα also promoted cell proliferation, extended cellular lifespan, and suppressed tumorigenesis (Acosta et al., 2008; Huang et al., 2002; Maier, Voulalas, Roeder, & Maciag, 1990).

In early stages of pregnancy, programed senescence is essential to optimize embryonic development. The senescence programs tissue remodeling by eliminating unwanted/transient cells or structures through recruiting immune factors during embryonic development (Banito & Lowe, 2013). Programed senescence in the embryo is mainly mediated by the P21 pathway in a P53‐independent manner whereas aging‐associated senescence is induced by activation of P53‐P21 or P16 pathways in response to DNA damage (Banito & Lowe, 2013). In this study, we found increases in both P53 and P21 in both aging and CXCL5‐treated young mouse embryos. In addition, PAI‐1, a downstream target of P53 (Kortlever, Higgins, & Bernards, 2006), was increased together with the elevation a SASP factor, IL6 in aging mouse blastocysts and CXCL5‐treated young blastocysts. Moreover, a previous study demonstrated that aging embryos contain more senescent cells as compared with the young one (Ge et al., 2019). These data suggest that aging‐associated senescence is mediated by CXCL5‐CXCR2 signaling pathway in aging embryos.

Aneuploidy is the most common abnormality in aging embryos causing failure of pregnancy (Franasiak et al., 2014). In this study, we demonstrated changes in gene expression profile between young and aging embryos at same morphological grade without consideration of aneuploidy. Using similar method without detection of aneuploidy, changes of gene expression profile by aging were reported in human and mouse oocytes resulting in different developmental potential (Grondahl et al., 2010; Hamatani et al., 2004). As shown in Table 1 and Figure 1a, although aging patients showed significant decreases in pregnancy rate, the pregnancy rate in young group was slightly lower than previously published data (Gardner et al., 2000). This might be caused by the accumulation of young patients with recurrent IVF failure in our university hospital. In contrast to the our microarray data showing deferentially expressed 820 genes in aging blastocysts obtained from patients at 38–45 years of age, other cells and tissues were reported to have lower number of differentially expressed genes during aging (e.g., up to 300 genes in brain, fat, muscular cells, and lymphocytes) (Berchtold et al., 2008; Cao, Gollapudi, Sharman, Jia, & Gupta, 2010; Glass et al., 2013; Welle, Brooks, Delehanty, Needler, & Thornton, 2003). In Gene ontology (GO) analysis, we found that six GO terms involved in biological process were identified as under or over‐represented. These GO groups were not specific in embryos and commonly detected in other type of aging cells (Grondahl et al., 2010; Shafiee, Asgari, Soltani, Larijani, & Heshmat, 2018). Based on microarray analysis demonstrating the highest expression of CXCL5 among major SASP factors in human embryos, we selected CXCL5 as a candidate for SASP factor in embryos. As compared with other SASP factors demonstrated to increase in aging cells/tissues (Freund, Orjalo, Desprez, & Campisi, 2010), the fold increases in *CXCL5* expression was high in aging blastocysts, suggesting its high possibility to work as a SASP factor.

We attempted to determine how CXCL5 affected the implantation ability by culturing young mice embryos with CXCL5. We found CXCL5 treatment of young embryos increased the expression of four aging biomarkers (Coppe et al., 2008; Goldstein, Moerman, Fujii, & Sobel, 1994; Kuilman et al., 2008), resulting in impairment of embryo implantation. Although the blastocyst attachment rate was not significantly decreased in the young embryos with CXCL5 treatment, the trophoblast spreading area was significantly reduced in a dose‐dependent manner. We also found a significant decrease in the number of trophoblastic giant cells, an indicator of implantation ability. Furthermore, CXCL5 treatment decreased the size of trophoblastic giant cells at the peripheral area of outgrowth accompanied with decrease in genes related to trophoblast migration (Zhu, Pang, & Yu, 2012), including matrix metalloproteinase (MMP) 1, 8, 9,10, and 15 in both CXCL5‐treated and aging embryos in our microarray data (GEO database: accession ID, GSE155477). These data suggest that CXCL5 suppressed blastocyst outgrowth through inhibition of trophoblast cell proliferation and migration, but not blastocyst attachment, during implantation as a SASP factor.

In this study, we demonstrated that suppression of CXCL5 signaling dramatically improved the implantation rate of aging mouse embryos. Thus, suppression of CXCL5 signaling during culture of embryos of middle‐aged women might improve the quality of aging embryos, leading to the development of new therapeutic methods. To anticipate future clinical applications, we evaluated the safety of endogenous CXCL5 signal suppression. We compared the gene expression in young mouse embryos, aging mouse embryos, and CXCL5 signaling‐suppressed aging mouse embryos. Following PCA analysis, the gene expression of CXCL5‐CXCR2 signaling‐suppressed aging embryos was found to be closer to young mouse embryos. These results suggest that suppression of CXCL5‐CXCR2 signaling could suppress aging‐related gene expression changes at the transcript level. Based on the microarray analysis, we found that suppression of CXCL5‐CXCR2 signaling enhanced two pathways related to cell proliferation, namely PI3 K signaling and RAS signaling, suggesting a potential role of these signaling pathways for improved implantation. Future characterization of affected genes following CXCL5‐CXCR2 axis suppression could reveal the exact mechanism underlying improved implantation in aging embryos. We also evaluated the effects of suppressing CXCL5‐CXCR2 signaling on both morphology and the weights of pups and placentas and found no adverse effects. In addition, we confirmed that suppression of CXCL5 signaling in young mouse embryos has no unwanted side effect in embryonic development and their potential for pregnancy, providing the basis for future use during culture of aging human embryos. Indeed, *Cxcl5* knockout mice showed no abnormality at the fetal stage and after birth, but the immune system was affected in adult mice (Balamayooran et al., 2012), suggesting suppression of CXCL5‐CXCR2 signaling during IVF unlikely affects embryonic development and the suppression procedure could be a treatment for aging human embryos.

In conclusion, we found increased CXCL5‐CXCR2 signaling as one of the causes of embryo aging. In addition, suppression of this signaling during embryo culture could improve pregnancy success in aging mice. Although we found CXCL5 as a SASP factor using human embryo transcriptome analyses, the studies of proof of concept have conducted using animal studies. Thus, future analysis to confirm the effect of CXCL5‐CXCR2 signaling suppression in human embryos is of interest and could be the basis for treating embryos in advanced aging patients. Also, monitoring CXCL5 expression in follicular cells and follicular fluid could be approaches to diagnose the levels of CXCL5 in individual oocyte/embryo obtained from patients who undergoing IVF‐ET treatment.

## 4 EXPERIMENTAL PROCEDURES

### 4.1 Human embryo preparation

Patients who underwent IVF at Akita University School of Medicine were targeted for this study. These patients were separated into two age groups (young group; 25–37 years of age and aging group; 38–45 years of age). Surplus cryopreserved blastocysts of these patients were used as samples. Informed consent for using these blastocysts was obtained from the patients. These blastocysts for each group were classified by Gardner's classification (Gardner et al., 2000), and morphologically high‐quality blastocysts which classified above 3BB according to Gardner criteria were thawed and used for microarray analysis. This study was approved by ethical committee of Akita University (No. 787) and Japan society of obstetrics and gynecology.

### 4.2 Animals

Two age group of female ICR mice (young; 3‐6 weeks of age and aging; 43‐53 weeks of age) were purchased from SLC Japan and maintained in a standard laboratory animal facility with controlled environment (temperature, 22; humidity, 55%–65%; and light cycle, 12 h interval). All animal experiments were approved by the Animal Care and Use Committee at St. Marianna University School of Medicine and International University of Health and Welfare School of Medicine.

### 4.3 Oocyte and embryo collection

Female ICR mice were super‐ovulated with intraperitoneal injection of 5 IU pregnant mare's serum gonadotropin (PMSG; Merck Millipore) followed 48 hours later by 5 IU human chorionic gonadotropin (hCG; ASKA Pharmaceutical). For oocyte retrieval, these mice were sacrificed at 14 h after hCG injection. Cumulus–oocyte complexes (COCs) were collected by dissecting the oviduct of mice and washed three times in modified HTF (mHTF; FUJIFILM Irvine Scientific) supplemented with 10% fetal bovine serum (FBS) (Cosmo Bio Co., Ltd.). For embryo collection, super‐ovulated mice were mated with male mice immediately after hCG treatment. At 18 h after hCG injection, zygotes were obtained by flashing the oviducts of mated mice. Cumulus–zygote complex were collected and washed three times in mHTF supplemented with 10% FBS, followed by cumulus removal in 80 IU/ml hyaluronidase (Sigma‐Aldrich). Zygotes were removed from the hyaluronidase immediately once the cumulus cells had detached and washed three times in mHTF before culture.

### 4.4 In vitro fertilization and embryo transfer

Collected COCs were placed in 100 μl drops of TYH medium (LSI Medience Corporation) covered by mineral oil in 35 mm culture dish (Thermo Fisher Scientific). These dishes were placed into CO_2_ incubator before sperm introduction for fertilization. After COCs collection, mature male ICR mice were immediately sacrificed and epididymis was removed for sperm preparation. This epididymis was punctured with a 21 G needle, and the sperms were squeezed into equilibrated 600 μl TYH medium. The sperms were stored in CO_2_ incubator for 10 min, and then, sperm suspension was added to the drops of TYH medium which contained COCs to give a final sperm concentration of 5.0 × 10^4^/ml. At 6 h after insemination, zygotic stage embryos were collected from the cumulus cells by gently pipetting and cultured in 30 μl of EmbryoMax^®^ KSOM mouse embryo medium (Merck Millipore) covered with mineral oil at 37°C and 5% CO_2_ in air up to specific stage embryos.

Female ICR mice (6–8 weeks of age) were mated with a vasectomized male ICR mouse to produce pseudo‐pregnant mice as a recipient for embryo transfer. Ten to sixteen blastocysts were transferred into the uterus of the day 3.5 pseudo‐pregnant recipients in each experimental group.

### 4.5 Regulation of CXCL5‐CXCR2 singling in mouse embryos

To assess the effect of CXCL5 on decline of embryo quality, CXCL5‐CXCR2 signaling was regulated in young and aging mouse embryos. Collected zygotes of young mouse were cultured in 30 μl drops of KSOM medium supplemented with different doses of CXCL5 peptide (Abcam, ab9803; 100, 300, and 1,000 nM) for activating CXCL5‐CXCR2 signal. Embryos were moved to fresh medium in every 48 h. The control groups for young embryos were cultured in KSOM without CXCL5 peptide or with 1,000 nM boiled‐CXCL5. Specificity of the effects of CXCL5 was confirmed by cotreatment with 10 μg/ml anti‐CXCL5 neutralizing antibody (Abcam, ab135203) and 10 nM CXCR2 selective non‐peptide antagonist (N‐(2‐hydroxy‐4‐nitrophenyl)‐*N*′‐(2‐bromophenyl)urea, Tocris Bioscience, SB225002). SB225002 is an antagonist of ^125^I‐IL‐8 binding to CXCR2 with an IC50 = 22 nM and shows >150‐fold selectivity over CXCR1 and other 7‐transmembrane receptors. SB225002 causes inhibition of IL‐8 and GROα‐mediated calcium mobilization in HL60 cells (White et al., 1998). In the aging mice, collected zygotes were cultured in 30 μl drops of KSOM medium supplemented with different doses of anti‐CXCL5 neutralizing antibody (0.1, 1.0, and 10 μg/ml) and/or CXCR2 selective antagonist (10 and 30 nM) for suppressing CXCL5‐CXCR2 signal. The control groups in aging embryos were cultured in KSOM without CXCL5 signal suppression. To rule out unwanted side effects of suppressing CXCL5‐CXCR2 signaling, young embryos were cultured in KSOM with anti‐CXCL5 neutralizing antibody (10 μg/ml) and/or CXCR2 selective antagonist (10 nM). At 96 hours after culture, blastocyst formation rate was determined. The mRNA levels of aging markers (*P16*, *P21*, *P53*, *Pai*‐*1*, and *Il*‐*6*) in young blastocysts with or without CXCL5 treatment and aging blastocysts at 96 h after culture were measured by real‐time quantitative RT‐PCR. These treated blastocysts were transferred to the uterus of pseudo‐pregnant recipients. All recipients were sacrificed at 15 days after blastocyst transfer. To assess the effect of CXCL5 signal on the implantation sites, the number of pups and the aborted fetuses were counted.

### 4.6 Blastocyst attachment and outgrowth assays

For further insight into negative effects of CXCL5 on embryo implantation, in vitro blastocyst attachment and outgrowth assays were performed as described (Kawamura et al., 2009). Young embryos at zygotic stage were cultured in 30 μl drops of KSOM medium supplemented with CXCL5 in each concentration up to expanded blastocyst stage. The embryos were then transferred to freshly prepared media every 48 h. Every expanded blastocyst was placed in M16 with 3% FBS in a chamber slide (Thermo Fisher scientific) and cultured for an additional 72 h in the presence of different doses of CXCL5. For negative controls, young embryos were cultured with or without 1,000 nM boiled‐CXCL5, whereas aging embryos cultured without CXCL5 were served as positive controls. Expanded blastocysts that adhered to the culture plate were designated as attached blastocysts. When trophoblast cells grew outward from the adhered blastocysts and the primary giant trophoblast cells became visible, these embryos were designated as blastocysts with outgrowth. The proportions of blastocysts undergoing attachment and outgrowth were estimated at 24 and 48 h, respectively (Kawamura et al., 2009). The proportions of hatched blastocysts showing attachment or outgrowth were used to estimate the implantation ability of the blastocysts in vitro. Furthermore, all outgrowth positive embryos were stained with Mouse anti‐α‐Tubulin‐Alexa 488 (Thermo Fisher scientific) and DAPI (Vector laboratories, Inc.) for measuring outgrowth area and counting the number of trophoblast giant cells, respectively.

### 4.7 DNA microarray

For analyzing the gene expression of human blastocysts, Agilent Whole Human V2 Genome Oligo Microarray platform 4 × 44 K (Agilent Technologies), which containing about 45,000 probe sets of oligo nucleotide probes was used. Besides, analyzing the gene expression of mouse embryos, SurePrint G3 Mouse GE v2 8 x 60 K Microarray (Agilent Technologies) was used. Five and 30 blastocysts were lysed using SuperAmp Lysis Buffer (Milteny Biotec) for human and mouse samples, respectively. Due to limited availability of human embryos and difficulty to collect large number of aging mouse embryos, we did not replicate the microarray analysis. mRNAs were extracted from lysate by using magnetic beads technology (Milteny biotec). Extracted mRNAs were amplified by SuperAmp RNA amplification kit (Milteny biotec) which is based on a single‐primer global PCR. Synthesis of cDNA was performed by using AffinityScript QPCR cDNA Synthesis Kit (Agilent) according to the manufacturer's instructions. The quantity of amplified mRNA‐derived cDNA was measured by the ND‐1000 Spectrophotometer (Thermo Fisher scientific). The integrity of the cDNA was checked by the Agilent 2100 Bioanalyzer platform (Agilent Technologies). Two hundred fifty ng of each cDNA samples was labelled with Cy3 dye (Agilent Technologies). The Cy3‐labeled cDNA was hybridized overnight at 65°C for 17 h to the microarray platform. These platforms were washed with the Agilent Gene Expression Wash Buffer 1 (Agilent Technologies) for 1 min at room temperature, and then washed with preheated Agilent Gene Expression Wash Buffer 2 (Agilent Technologies) for 1 min at 37°C Finally, these platforms were washed with acetonitrile (Agilent Technologies) and scanned in the Agilent Microarray Scanner system (Agilent Technologies) detecting fluorescence signals. Fluorescence images were read out using the Agilent Feature extraction software (Agilent Technologies).

### 4.8 Microarray data analysis

Microarray data were analyzed by using Genespring GX 10.7 (Agilent Technologies). The intensity of genes was normalized and compared in each group. Probe sets were defined as being differentially expressed genes if the P‐value below 0.01 and the Log_2_ fold change above five were confirmed. Microarray data of human and mouse blastocysts were deposited into Gene Expression Omnibus (GEO) repository and are accessible through GEO accession number GSE155476 and GES155477, respectively. KEGG (Kyoto Encyclopedia of Genes and Genomes) pathway analysis was carried out to identify the enrichment signaling pathways in the CXCL5‐suppressed aging blastocysts. *p*‐value <0.05 indicated the significant enriched signaling pathways. The principal component analysis (PCA) was also performed by R (v. 3.5.3).

### 4.9 Validation of microarray results by real‐time quantitative RT‐PCR

To confirm the results of microarray analysis for human blastocysts, expression of *CXCL5* was measured using real‐time quantitative RT‐PCR. Some amounts of total RNA prepared for microarray analysis were reverse transcribed into cDNA using a Sensicript RT Kit (Qiagen). Real‐time quantitative RT‐PCR was performed using iTaq SYBR Green SuperMix (Bio‐Rad Laboratories, Inc.) on a Smart Cycler TD System (Cepheid) as follows: 15 min at 95, followed by 45 cycles of 15 s at 95°C and 60 s at 60°C. Relative *CXCL5* mRNA levels were calculated based on the Ct values and normalized by *HISTON H2A*. Primers are shown in Table S1.

### 4.10 Real‐time quantitative RT‐PCR

Total RNA was extracted from oocytes and embryos at various developmental stage of young and aging mice using a RNeasy micro kit (Qiagen) according to the manufacturer's instructions. RNeasy mini kit (Qiagen) was used to isolate total RNA from tissue samples. cDNA synthesis was performed by using PrimeScript II 1st strand cDNA Synthesis Kit (Takara Bio, Inc.) according to the manufacturer's instructions. Real‐time quantitative RT‐PCR reaction was carried out on a light cycler 96 (Roshe Diagnostic) using with LightCycler 480 SYBR Green I Master (Roshe Diagnostic). Relative mRNA levels were calculated based on the C*_t_* values and normalized by Histon H2a for oocyte and embryo and β‐actin for tissue samples. The primer sequences are listed in Table S1.

### 4.11 Statistical analysis

Statistical analysis was performed using statistical software package R (v. 3.5.3). Categoric data between two groups were compared by the Mann–Whitney *U* test. Quantitative data between two groups were compared by Student's *t* test. Multiple analysis was performed using the Dunnett test. *p*‐value of <0.05 was considered statistically significant unless specified in the materials and methods.

## CONFLICT OF INTEREST

The authors confirm that they have no conflict of interest.

## AUTHOR CONTRIBUTIONS

K.K. designed the research and wrote the manuscript. Y.K. designed and performed the experiments, analyzed the data, and wrote the manuscript. I.K. and K.M. contributed to the data analysis. Y.S. and N.O. performed the experiments and contributed to the data analysis.

## Supporting information

Fig S1

Table S1

## Data Availability

The data that support the findings of this study are available from the corresponding author upon reasonable request.
