# Supplementary figures and images for: CXCL5‐CXCR2 signaling is a senescence‐associated secretory phenotype in preimplantation embryos
Source: Aging Cell. 2020 Sep 22;19(10):e13240. doi: 10.1111/acel.13240 (PMC7576282; doi:10.1111/acel.13240)

Figure\_S1

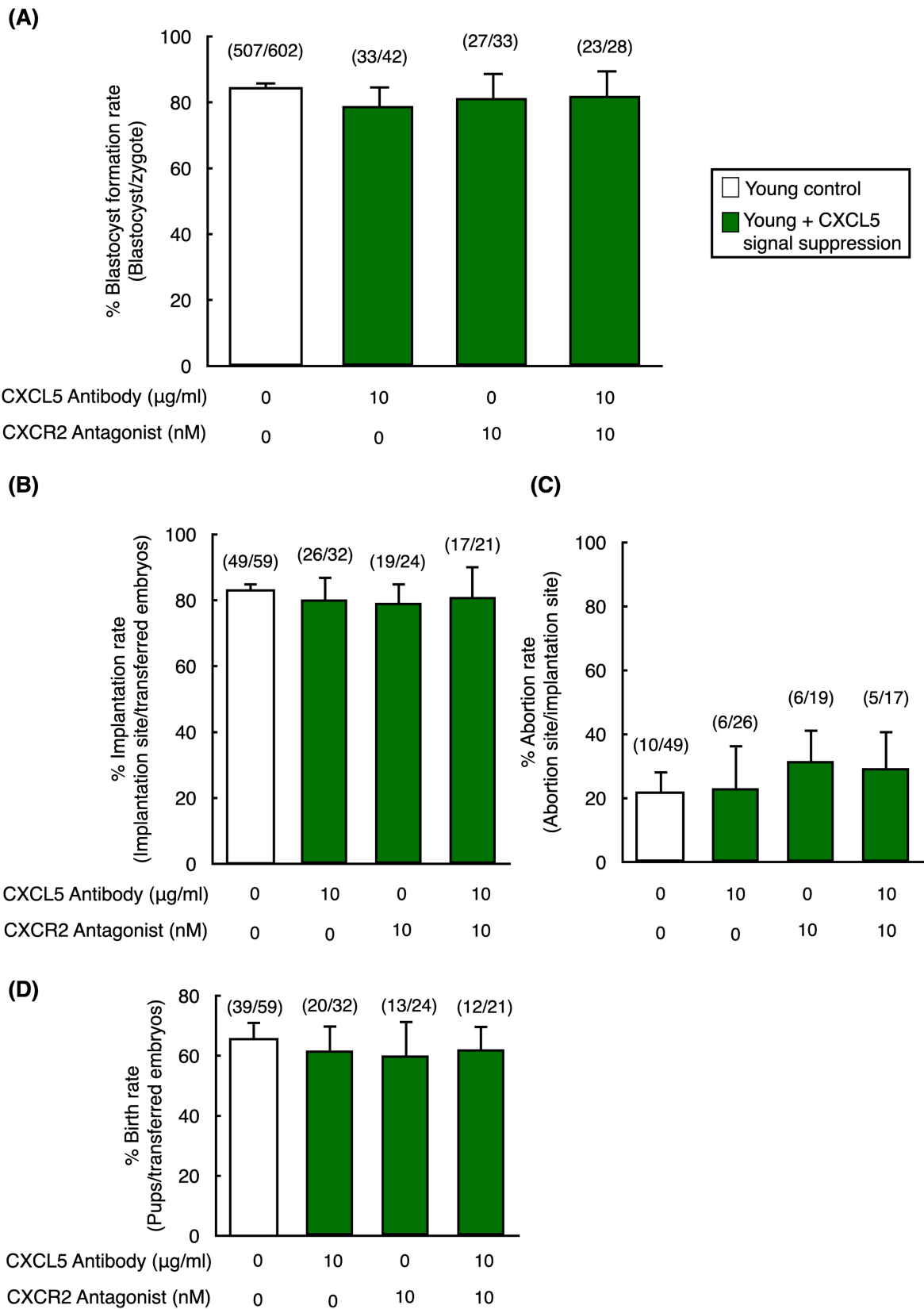

Supplement: Supplementary file 1 — Fig S1 [file ACEL-19-e13240-s001.pdf]
