# Supplementary material for: CXCL5‐CXCR2 signaling is a senescence‐associated secretory phenotype in preimplantation embryos
Source: Aging Cell. 2020 Sep 22;19(10):e13240. doi: 10.1111/acel.13240 (PMC7576282; doi:10.1111/acel.13240)
Supplement: Supplementary file 2 — Table S1 [file ACEL-19-e13240-s002.pdf]

Table S1. Sequences for the primers used for quantitative RT-PCR analysis.

Species	*Gene*	Oligonucleotide Sequence (5' → 3')	
Mouse	*Cxcl5*	Forward	GCATTTCTGCTGCTGTTCACACT
		Reverse	GGTTAAGCAAACACAGCGTAGCT
	Cxcr2	Forward	TCCTAACACTAGACCCCAAACACTC
		Reverse	TTTCTCTCCTCCACCTCTTCCTT
	P16	Forward	GCGTGTCTAGCATGTGGCTTT
		Reverse	TCCTTCTGCTCCCTCCCTCT
	P21	Forward	AATACCGTGGGTGTCAAAGCA
		Reverse	AGGGAGGGAGCCACAATACA
	P53	Forward	GATGCCCATGCTACAGAGGAG
		Reverse	ACAGAAAAGGGGAGGGATGAA
	Pai-1	Forward	TTCCTCTCACTTCCACCCAAA
		Reverse	AGCAACAGCAACAGAAACAACAC
	Il-6	Forward	CACCAAGAACGATAGTCAATTCCA
		Reverse	TCACCAGCATCAGTCCCAAG
	Histon H2a	Forward	GCTTGCTATACGTGGAGATGAAGA
		Reverse	AGCGATTTGTGGATGTGTGG
	β-actin	Forward	AGGGAGGGAGCCACAATACA
		Reverse	AGGGAGGGAGCCACAATACA
Human	*CXCL5*	Forward	CCACTATGAGCCTCCTGTCC
		Reverse	CAACGCAGCTCTCTCAACAC
	HISTON H2A	Forward	GCTTGCTATACGTGGAGATGAAGA
		Reverse	AGCGATTTGTGGATGTGTGG
